# RIP3 blockade prevents immune-mediated hepatitis through a myeloid-derived suppressor cell dependent mechanism

**DOI:** 10.7150/ijbs.65402

**Published:** 2022-01-01

**Authors:** Man Liu, Hongxia Zhang, Lu Zhang, Xin Liu, Simin Zhou, Xiaoyi Wang, Weilong Zhong, Jie Zhang, Bangmao Wang, Jingwen Zhao, Lu Zhou

**Affiliations:** 1 Department of Gastroenterology and Hepatology, General Hospital, Tianjin Medical University, Tianjin, China.; 2 Department of Physiology and Pathophysiology, Tianjin Medical University, Tianjin, China.; 3 Department of Gastroenterology and Hepatology, People's Hospital of Hetian District, Xinjiang Uygur Autonomous Region, China.

**Keywords:** immune-mediated hepatitis, receptor-interacting protein kinase 3, myeloid-derived suppressor cells, cytokines and chemokines, glucocorticoid treatment

## Abstract

Autoimmune hepatitis (AIH) is an immune-mediated chronic inflammatory liver disease, and its pathogenesis is not fully understood. Our previous study discovered that receptor interacting protein kinase 3 (RIP3) is correlated with serum transaminase levels in AIH patients. However, its role and underlying mechanism in AIH are poorly understood. Here, we detected the increased expression and activation of RIP3 in livers of patients and animal models with AIH. The inhibition of RIP3 kinase by GSK872 prevented concanavalin A (ConA)-induced immune-mediated hepatitis (IMH) by reduced hepatic proinflammatory cytokines and immune cells including Th17 cells and macrophages. Further experiments revealed that RIP3 inhibition resulted in an increase in CD11b^+^Gr1^+^ myeloid-derived suppressor cells (MDSCs) with immunoregulatory properties in the liver, spleen, and peripheral blood. Moreover, the depletion of Gr-1^+^ MDSCs abrogated the protective effect and immune suppression function of GSK872 in ConA-induced IMH. Altogether, our data demonstrate that RIP3 blockade prevents ConA-induced IMH through promoting MDSCs infiltration. Inhibition of RIP3 kinase may be a novel therapeutic avenue for AIH treatment.

## Introduction

Autoimmune hepatitis (AIH) is a progressive immune-mediated liver disorder characterized by elevated serum transaminases, interface hepatitis, the presence of circulating autoantibodies, and hypergammaglobulinemia 1-4. Originally AIH was believed to be a disease of young women. Currently it is known that AIH can present at all ages, moreover, the rate of cirrhosis at diagnosis of AIH was similar at all ages 5. The etiology of AIH is incompletely understood, and clinical treatments rely on chronic administration of high doses of prednisolone (occasionally associated with an immunosuppressant such as azathioprine). However, these agents, particularly corticosteroids, can be associated with important side effects, such as metabolic syndrome with its sequelae and osteoporosis 6-8. Thus, further understanding of the disease's cellular and molecular mechanisms may assist in identifying better treatment modalities, aid the limitation of adverse effects from therapy, and improve prognostication.

Immune response imbalance is one of the major hallmarks of AIH. Excessive activation of macrophages and imbalance between T regulatory cells (Tregs) and T effector cells contribute to the perpetuation of liver damage in AIH 9-12. Concanavalin A (ConA)-induced hepatitis is an appropriate animal model for immune-mediated liver injury in human AIH 13-15. A growing evidence supports the critical role of inflammatory cells, including T cells, natural killer (NK) cells, NK T-cells, and monocytes/macrophages, in the pathophysiology of AIH in animal and human research 16, 17. Thus, if the function of these cells can be regulated and inhibited, then liver inflammation can be attenuated.

Myeloid-derived suppressor cells (MDSCs) have emerged as major regulators of inflammation and immune function in a variety of pathological conditions 18-20. MDSCs can repress the activation of T cells, B cells, macrophages, and NK cells but activate Tregs 21-23. Recent findings suggest that dampening MDSC suppressor function is an attractive strategy for optimizing cancer immunotherapy and contributing to enhancing the sensitivity of anti-tumor immunotherapy 19, 24, 25. Conversely, enhancing or sustaining MDSCs suppressive function would be beneficial in inhibiting inflammation and immune disorders 26. Accumulating evidence indicates that MDSCs can protect against liver injury in AIH patients and experimental immune-mediated hepatitis (IMH) 27-29. Therefore, the regulation of MDSCs is a promising direction to ameliorate immune hepatitis. However, the methods to successfully maintain MDSCs in the desired suppressive state in AIH remain poorly defined.

Receptor interacting protein kinase 3 (RIP3) is a molecular switch between tumor necrosis factor (TNF)-induced apoptosis and necrosis 30. We previously showed that RIP3 signaling is highly correlated with serum transaminase levels in patients with AIH, indicating that RIP3 signal plays important roles in the pathogenesis of the disease 31. On the other hand, RIP3 also targets and delivers negative signals to MDSCs recruitment in tumor research 32, 33. However, its role in AIH remains largely unknown. Therefore, in this present study, we investigated the role of RIP3 in IMH. Activation of RIP3 were detected readily in livers of patients and animal models with AIH. RIP3 blockade protected mice against IMH through a mechanism mediated by CD11b^+^Gr-1^+^ MDSCs. In addition, the expression of RIP3 in livers of IMH mice was significantly decreased by glucocorticoid treatment, suggesting that RIP3 is a potential target for the treatment of AIH.

## Materials and methods

### Participants

A total of eight AIH patients and five controls with hepatic cyst were recruited from the Department of Gastroenterology and Hepatology in Tianjin Medical University General Hospital between January 2019 and August 2019. The enrolled AIH patients fulfilled the 1999 revised International Autoimmune Hepatitis Group (IAIHG) classification criteria of AIH 34. No patients were treated with corticosteroids. Sex and age-matched controls fulfilled the following inclusion criteria: (1) cyst diameter <4 cm and cyst fluid is clear, (2) normal ranges of liver function test, (3) normal histopathology of liver tissue next to the cyst, (4) an absence of hepatitis B/C virus antigen. Liver biopsy specimens was obtained from all participants. This study was approved by the Ethics Committee of Tianjin Medical University General Hospital, and all participants provided signed informed consents.

### Animal experiments

Female C57BL/6 mice (6-week-old) were obtained from the Institute of Laboratory Animal Science, Chinese Academy of Medical Sciences & Peking Union Medical College and maintained under specific pathogen-free environment. All the mice were acclimatized for 1 week and then randomized into four groups (n=6-7 per group). According to the previous studies 35, 36, mice were intravenously (i.v.) injected with ConA (15 mg/kg; Solarbio, Beijing, China) for 12 h to establish a mouse model of IMH. To evaluate whether RIP3 blockade prevents IMH, the mice were i.p. injected with GSK872 (1 mg/kg; Selleck, USA), dexamethasone (DEX, 1 mg/kg; Sigma, USA), or an equal volume of vehicle (phosphate-buffered saline (PBS) containing 2% dimethyl sulfoxide) 1 h before intravenous injection of ConA (15 mg/kg; Solarbio, Beijing, China). Control mice were given PBS alone. Blood samples were collected 12 h after ConA administration by retro-orbital bleeding, and the mice were sacrificed. Livers and spleens were harvested.

For MDSCs depletion, mice were i.p. injected with a depleting anti-mouse Gr-1 antibody (250 µg/mouse; BioXCell, USA) or an isotype control (250 µg/mouse; BioXCell, USA) 36 h before ConA administration 37, 38. GSK872 were i.p. given 1 h before ConA treatment. All animal studies were performed in accordance with the National Institutes of Health Guide for the Care and Use of Laboratory Animals. The animal use protocol has been reviewed and approved by the Animal Ethical and Welfare Committee of Tianjin Medical University, Approval No. IRB2020-WZ-175.

### Assay for serum aminotransferase activity

Alanine aminotransferase (ALT) and aspartate aminotransferase (AST) activities were measured by standard procedures at the Institute of Clinical Chemistry of the Tianjin Medical University General hospital.

### Analysis of liver index and spleen index

Liver index was calculated as (liver weight (g)/body weight (g)) *100%. Spleen index was calculated as (spleen weight (g)/body weight (g)) *100%.

### Real-time quantitative polymerase chain reaction (RT-qPCR)

Total RNA was extracted from liver using TRIzol reagent (Invitrogen, USA). cDNA was synthesized using TIANScript RT Kit (Tiangen, Beijing, China) following the manufacturer's protocol. qPCR analysis was performed using SYBR Green PCR Master Mix (ThermoScientific) and specific primers (Genewiz, Beijing, China). Table 1 shows the oligonucleotide primers for target genes. All values were normalized to the levels of Glyceraldehyde-3-phosphate dehydrogenase (GAPDH). The relative mRNA expression of the target gene was calculated by standard ΔΔCt method.

### Immunofluorescence

Liver tissues were cut, deparaffinized, dehydrated, and incubated with specific antibodies against p-RIP3 (Abcam, USA), p-MLKL (Abcam, USA) and CD68 (Abcam, USA) overnight at 4 °C. Subsequently, the sections were washed thrice with 1 × PBS and then incubated with fluorochrome-conjugated secondary antibody for 60 min at room temperature in the dark. 4, 6-Diamidino-2-phenylindole was finally applied to dye the nucleus. A fluorescence microscope DM5000 B (Leika, Germany) was used for analysis.

### Histopathology

The formalin-fixed liver tissues were routinely dehydrated, equilibrated, embedded in paraffin, and then cut into sections. Paraffin sections (2 μm) were stained with H&E and examined under light microscopy to determine the level of inflammation.

### Western blot analysis

The liver tissues were lysed using RIPA lysis buffer containing protease inhibitors and phosphatase inhibitor (Solarbio, Beijing, China). Proteins were separated using sodium dodecyl sulphate-polyacrylamide gel electrophoresis system and transferred onto polyvinylidene difluoride membranes. The membranes were incubated overnight at 4 °C with specific primary antibodies: anti-RIP3 Ab (Santa Cruz), anti-MLKL Ab (CST, USA), anti-phospho-RIP3 Ab (CST, USA), anti-phospho-MLKL Ab (CST, USA), and anti-GAPDH Ab (CST, USA). After washing in Tris-buffered saline and Tween 20, the membranes were incubated with horseradish peroxidase-conjugated secondary antibodies (CST, USA). Visualization was achieved using an ECL plus detection system (Solarbio, Beijing, China). ImageJ software was used for quantified analysis.

### Mononuclear cell isolation for flow cytometry

HMNCs were isolated by the method of Diao et al. 27. with minor modifications. Briefly, mouse livers were minced and digested with collagenase Ⅳ (Solarbio, Beijing, China) for 45 min at 37 °C. The cell suspensions were filtered with 70 µm nylon cell strainers (Solarbio, Beijing, China) and centrifuged at 300 g for 5 min. The pellet was washed with PBS and resuspended in 40% Percoll. The cell suspension was gently overlaid onto 70% Percoll and centrifuged for 20 min at 750 g. Purified HMNCs were collected from the interphase and washed twice in PBS. Spleens and peripheral blood from mice were collected, grinded, and filtered. After depleting erythrocytes, purified mononuclear cells were collected for further flow cytometry analysis.

### Flow Cytometric Analysis

The mononuclear cells were stained with zombie NIR™ (BioLegend, USA) to distinguish live cells from dead cells, and incubated with Fc block (BioLegend, USA) for 10 min at room temperature. Then cells were washed, and incubated with the following fluorescently conjugated mAbs (BD Bioscience, USA): fluorescein isothiocyanate (FITC) anti-CD45, PEcy7 anti-CD11b, AF®647 anti-F4/80, PE anti-Gr-1, BV421 anti-CXCR2, for 45 min at 4 °C in the dark. In all experiments, appropriate isotype control IgGs were used. FACSverse flow cytometer (BD Bioscience, USA) and FlowJo software were used for analysis.

For CD4^+^IL-17^+^ T cells, cells were stimulated by cell activation cocktail (BioLegend, USA) in incubator (5% CO_2_, 37 °C) for 6 h. Subsequently, using zombie NIR™ to distinguish live cells from dead cells, and incubated with Fc block. Cells were incubated with FITC-anti-CD4 (BD Bioscience, USA), and then cells were labeled with PE-anti-IL-17A (BD Bioscience, USA) after fixation and permeabilization.

For CD4^+^CD25^+^ Foxp3^+^ cells, cells were stained with zombie NIR™ to distinguish live cells from dead cells and then incubated with Fc block. Subsequently, cells were labeled with FITC-anti-CD4 (BD Bioscience, USA) and PE/Cyanine7-anti-CD25 (BD Bioscience, USA). Then cells were labeled with Alexa Fluor647-Foxp3 (BD Bioscience, USA) after fixation and permeabilization.

### T cell proliferation assay

Hepatic CD11b^+^Gr-1^+^ MDSCs and splenic CD4^+^T cells from different groups were purified by Cell Isolation Kit II (Miltenyi Biotec, Germany) with >90% purity according to the manufacturer's instruction. CD4^+^Tcells were labeled with 2 µM CFSE (Invitrogen, USA). Diverse MDSCs were co-cultured with CFSE-labeled CD4^+^T cells at 1:2 ratio stimulated with anti-CD3/CD28 activating antibodies (Miltenyi Biotec, Germany). And T cell proliferation was determined after 72 h of coculture by flow cytometry.

### Statistical analysis

Data are expressed as mean ± standard deviation (SD). The data for two-group comparisons were assessed using two-tailed Student's *t* test (parametric data). Differences among more than two groups were tested by one-way ANOVA. *P* < 0.05 was considered statistically significant. Statistical analysis was performed on SPSS 22.0.

## Results

### Activation of RIP3 in liver tissues of AIH patients and IMH mice

**Figure 1:**
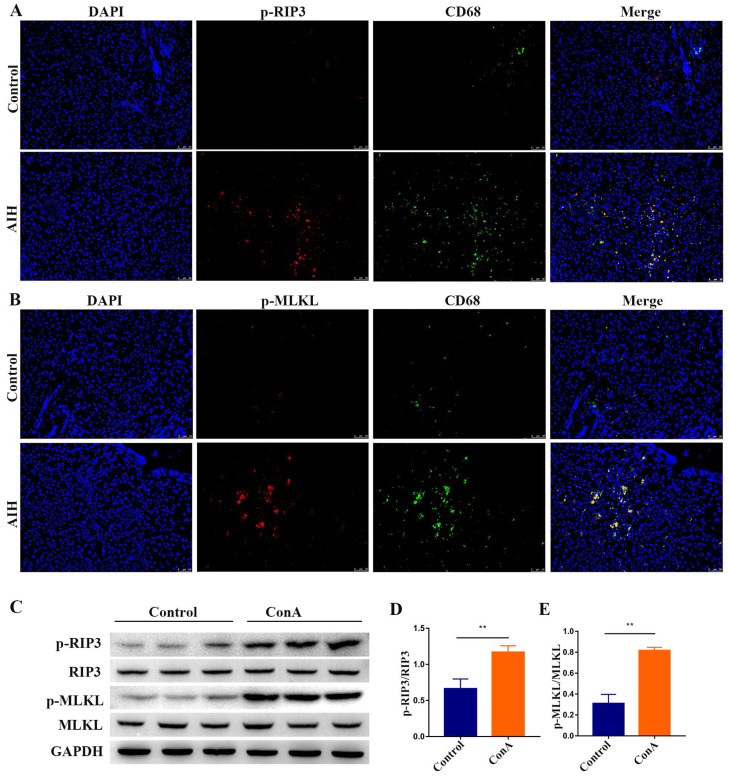
** Activation of RIP3 signaling in the livers of AIH patients and IMH mice.** Representative p-RIP3 **(A)** and p-MLKL** (B)** stained liver biopsy sections from control (hepatic cyst, n=5) and AIH patients (n=8). Original magnification, ×200; scale bars, 50 μm. **(C)** Mice, which were injected intravenously (i.v.) with ConA or vehicle (n=7 per group), were sacrificed at the indicated time points, and livers were collected and prepared for extraction of total proteins. Representative Western blots showing the expression levels of p-RIP3, RIP3, p-MLKL, and MLKL. GAPDH was used as loading control. Quantitative increases in p-RIP3 **(D)** and p-MLKL **(E)**. All the values are shown as mean ± SD; ns, not significant; *P < 0.05; **P < 0.01.

Initially, we analyzed the expression of p-RIP3 and p-mixed lineage kinase domain‑like protein (MLKL), which are the phosphorylated active forms of RIP3 and its downstream target MLKL, respectively, in paraffin-embedded liver sections of patients with AIH and controls by immunofluorescence. As shown in **Figure 1A-B**, p-RIP3-positive cells and p-MLKL-positive cells markedly increased in the liver sections from AIH patients, particularly in CD68-positive macrophages, but staining was virtually absent in the control livers, thus indicating that RIP3 signaling is activated during AIH.

Livers from ConA-treated mice were analyzed for evidence of RIP3 activation to corroborate the above results. As shown in **Figure 1C-E**, Western blotting analysis of liver extracts showed that the expressions of p-RIP3 and p-MLKL were significantly higher in mice treated with ConA in comparison with the controls, further proving that IMH associates with the activation of RIP3 signaling.

### RIP3 blockade protects mice against immune-mediated liver injury

**Figure 2:**
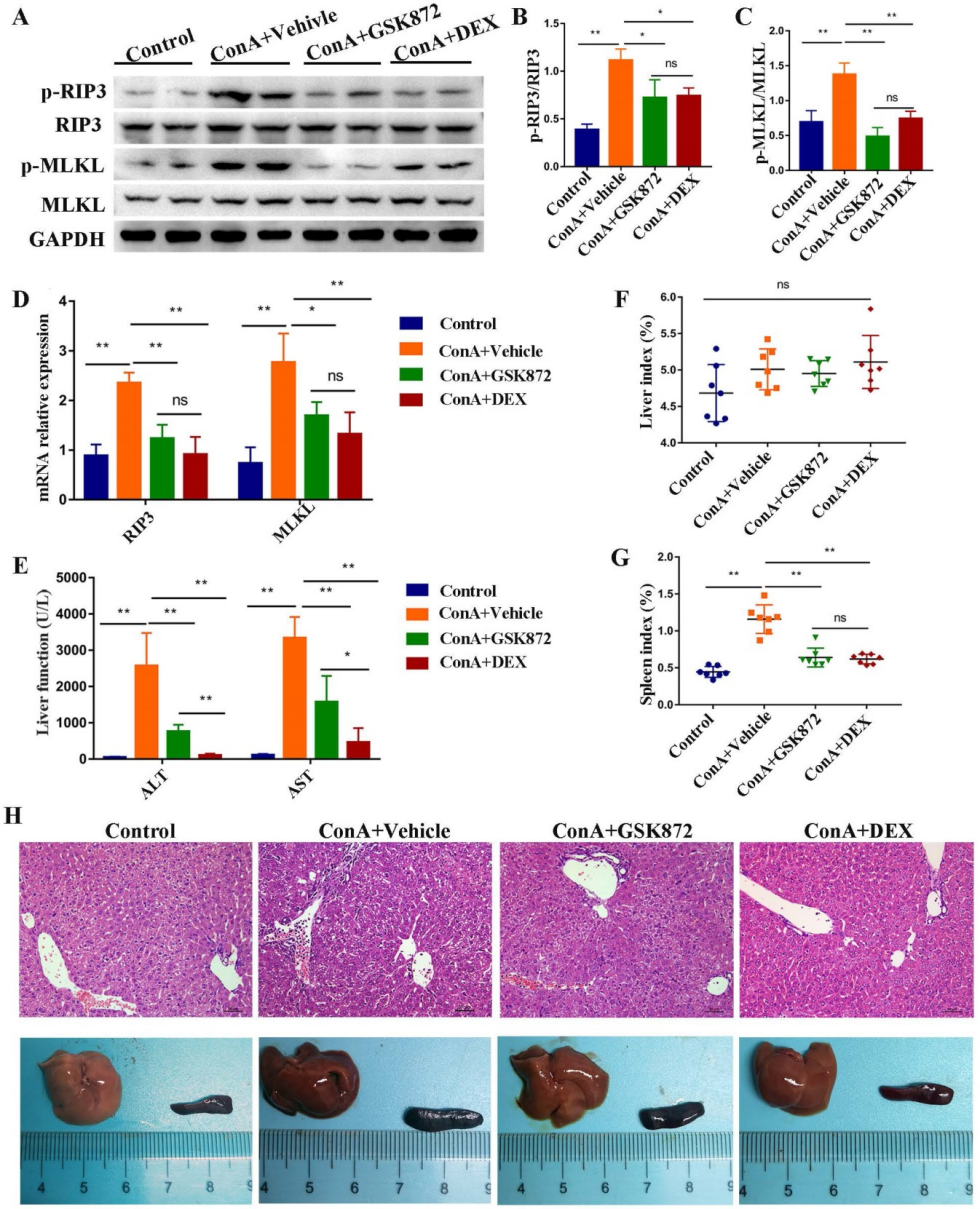
** RIP3 blockade protects mice against immune-mediated liver injury.** Mice were injected i.p. with GSK872 or DEX or vehicle 1 h before treatment with ConA. Control mice were given PBS alone (n=7 per group).** (A)** Immunoblot analyses of p-RIP3, RIP3, p-MLKL, and MLKL protein levels in the livers. GAPDH served as loading control. Quantitative expressions of p-RIP3 **(B)** and p-MLKL **(C)**. **(D)** RIP3 and MLKL mRNA levels were assessed by RT-qPCR. Gene expression was normalized to GAPDH levels. **(E)** Serum levels of ALT and AST were measured 12 h after ConA treatment. The liver index **(F)** and spleen index** (G)** were determined 12 h after ConA treatment. **(H)** Representative photomicrographs (H&E staining; original magnification 200×; scale bars, 50 μm; up) and perusal images (down) of livers in four groups. All the values are shown as mean ± SD. ns, not significant; *P < 0.05, **P < 0.01.

Next, we examined whether blockading RIP3 can prevent IMH. We took advantage of GSK872, a highly potent and selective RIP3 kinase inhibitor. Mice were pretreated intraperitoneally (i.p.) with GSK872, DEX, or vehicle 1 h before ConA administration and were sacrificed 12 h later. As shown in **Figure 2A-C**, induction of IMH by ConA was followed by elevated p-RIP3 and p-MLKL activities in the livers. Pretreatment of mice with GSK872 significantly reduced the expressions of p-RIP3 and p-MLKL, indicating that RIP3 activation can be effectively inhibited by RIP3 inhibitor. Notably, the activation of p-RIP3 and p-MLKL induced by ConA administration can be inhibited by DEX treatment, suggesting that RIP3 signaling is targeted by immunosuppressant DEX. Likewise, the overexpression of RIP3 and MLKL mRNA induced by ConA administration were abolished by GSK872 or DEX pretreatment** (Figure 2D)**.

The serum levels of ALT and AST increased in ConA-injected mice were significantly reduced by GSK872 or DEX pretreatment **(Figure 2E)**. Although the liver index was not significantly altered, the spleen index and size increased in ConA-injected mice, and the results can be reversed by GSK872 or DEX pretreatment **(Figure 2F-H)**. Moreover, hematoxylin and eosin (H&E) staining of liver sections showed reduced infiltration of inflammatory cells and minimal necrosis in mice injected with ConA and receiving GSK872 or DEX **(Figure 2H)**. Collectively, these data show the effectiveness of inhibiting RIP3 in preventing IMH, consistent with the therapeutic effects of DEX.

### RIP3 blockade reduces hepatic levels of pro-inflammatory cytokines and immune cells during IMH

**Figure 3:**
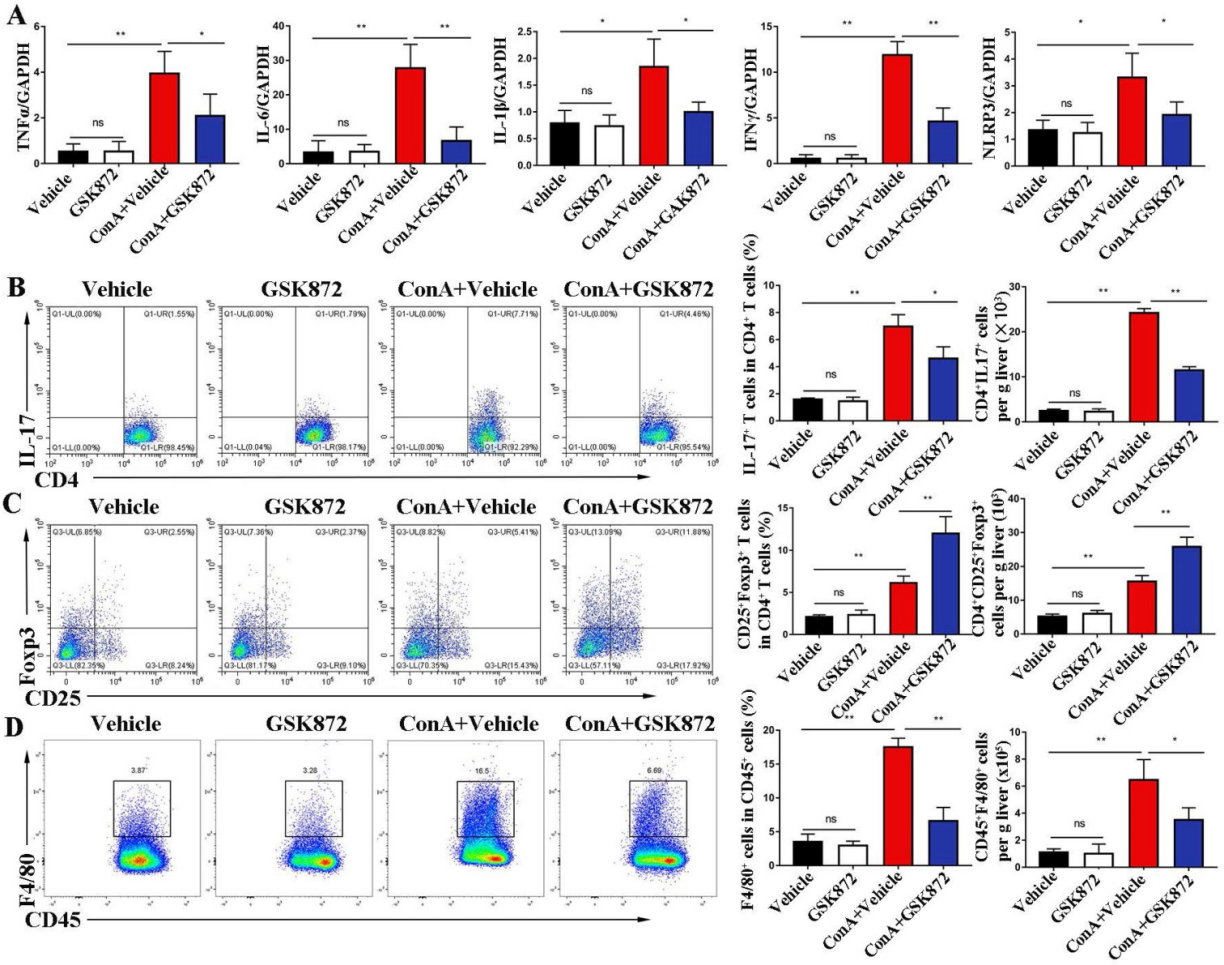
** RIP3 blockade inhibits ConA-induced hepatic immune activation. (A)** Expression levels of TNF-α, IL-6, IL-1β, IFNγ, and NLRP3 in livers were analyzed by RT-qPCR. Gene expression was normalized to GAPDH levels. Fractions of CD4^+^IL-17^+^ cells **(B)** and CD4^+^CD25^+^ Foxp3^+^ cells **(C)** in liver CD4^+^ T cells were assessed by flow cytometry. Representative scatter plots (left) are presented, and the histogram (right) represents the statistical analysis of the percentages of gated in liver CD4^+^ T cells. **(D)** Flow cytometry analysis of CD45^+^F4/80^+^ macrophages in CD45^+^ HMNCs (left). The histogram (right) represents the statistical analysis of the percentages of CD45^+^F4/80^+^ macrophages in CD45^+^ HMNCs. All the values are shown as mean ± SD. ns, not significant; *P < 0.05, **P < 0.01.

To investigate the mechanism by which RIP3 blockade prevents immune-induced liver injury, we examined the proinflammatory cytokines and immune cells involved in IMH. As shown in **Figure 3A**, the induction of liver damage by ConA was accompanied by significant upregulation of TNFα, interleukin (IL)-6, IL-1β, interferon (IFN) γ, and NLR family pyrin domain containing 3 (NLRP3), which can be attenuated by pretreatment with GSK872. Next, we determined whether RIP3 blockade changes immune cell infiltration in the livers. Flow cytometry analysis revealed that ConA inducement increased the accumulation of CD4^+^IL-17^+^ Th17 cells, CD4^+^CD25^+^Foxp3^+^ Tregs and CD45^+^F4/80^+^ macrophages **(Figure 3B-D)**. Pretreatment with GSK872 was associated with decreased CD4^+^IL-17^+^ Th17 cells and CD45^+^F4/80^+^ macrophages and increased infiltration of CD4^+^CD25^+^Foxp3^+^ Tregs. Altogether, these data support that RIP3 blockade inhibits the production of inflammatory cytokines and infiltration of proinflammatory immune cells but promotes the accumulation of CD4^+^CD25^+^ Foxp3^+^ Tregs with immunoregulatory properties in livers of IMH mice.

### RIP3 blockade promotes accumulation of CD11b^+^Gr-1^+^ MDSCs in the liver, spleen, and peripheral blood of IMH mice

**Figure 4:**
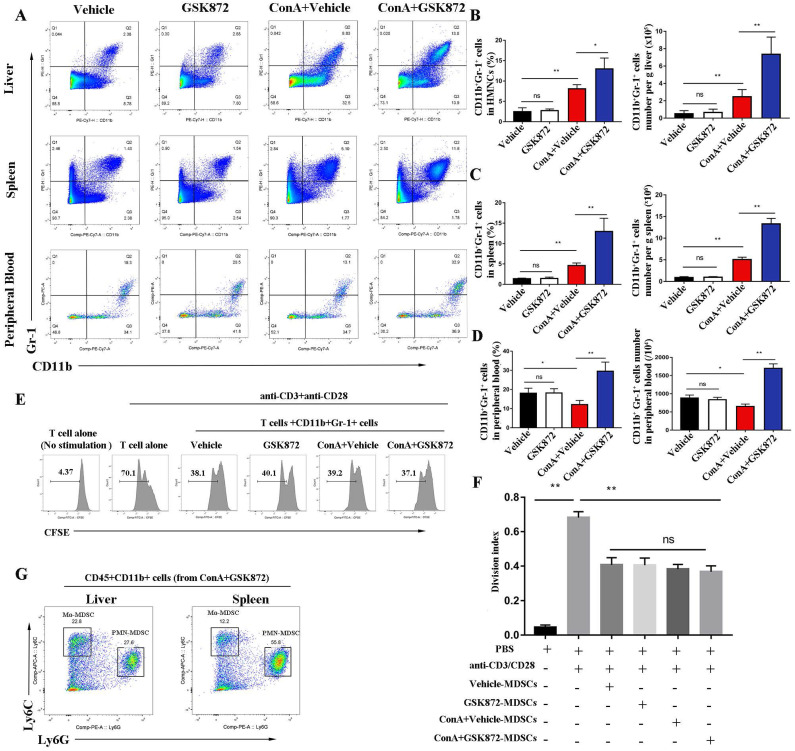
**RIP3 blockade induces the accumulation of CD11b+Gr-1+ MDSCs in IMH mice. (A)** Flow cytometry analysis of CD11b^+^Gr-1^+^ MDSCs in the liver, spleen, and peripheral blood. The statistical analysis of the percentages and absolute cell number of CD11b^+^Gr-1^+^ MDSCs in the liver** (B)**, spleen **(C)**, and peripheral blood** (D)**. **(E)** Flow cytometric analysis of proliferation of native splenic CD4^+^ T cell cocultured with diverse MDSCs in the presence of anti-CD3/CD28 for 72 h. **(F)** The frequencies of the proliferating CD4+ T cells. **(G)** The percentage of Ly6G^hi^ghLy6C^lo^ (PMN-MDSCs) and Ly6G^lo^Ly6C^hi^ (Mo-MDSCs) in CD45^+^CD11b^+^ HMNCs of ConA+GSK872 treated-mice. All the values are shown as mean ± SD. ns, not significant; *P < 0.05, **P < 0.01.

CD11b^+^Gr-1^+^ cells, termed as MDSCs, are a heterogeneous subset of myeloid cells with a potent immune-suppressive ability 39. To evaluate whether RIP3 blockade induces the accumulation of MDSCs to prevent ConA-induced hepatitis, we employed flow cytometry to detect the frequency of CD11b^+^Gr-1^+^ MDSCs in mice livers, spleens, and peripheral blood. Compared with the controls, the frequency of CD11b^+^Gr-1^+^ MDSCs increased in livers and spleens but reduced in the peripheral blood of ConA-injected mice **(Figure 4A-D)**. The upregulation of frequency of CD11b^+^Gr-1^+^ MDSCs in livers and spleens induced by ConA was further increased by pre-treatment with GSK872. Moreover, the populations of CD11b^+^Gr-1^+^ MDSCs in peripheral blood of ConA-injected mice were strongly upregulated by GSK872 pretreatment. Another RIP3 inhibition GSK843 confirmed the effect of RIP3 blockade on CD11b^+^Gr1^+^ MDSCs accumulation in ConA treated-mice **(Figure S1)**. Altogether, these data demonstrate that RIP3 blockade promotes MDSCs accumulation in ConA-induced hepatitis.

Next, to examine whether they have the immunosuppressive properties, we isolated hepatic CD11b^+^Gr-1^+^ MDSCs from each group of mice and cocultured them with splenic CD4^+^ T cells isolated from wild mice in the presence of anti-CD3 and anti-CD28 antibodies. The results showed that CD11b^+^Gr-1^+^ cells isolated from each group strongly suppressed the proliferation of CD4^+^ T cells compared with when T cells alone were activated, which confirmed their immunosuppressive capacities **(Figure 4E-F)**. However, there was no difference in the ability of hepatic MDSCs from different groups to inhibit T cell proliferation, demonstrating that RIP3 inhibition does not directly influence MDSCs suppressive function but rather increases the immunosuppressive environment via MDSCs expansion during IMH.

Two major subsets of mice MDSCs have been identified based on the expression of CD11b, Ly6G and Ly6C antigens 18. Polymorphonuclear MDSCs (PMN-MDSCs) expressing CD11b^+^Ly6G^hi^Ly6C^lo^ and monocytic MDSCs (Mo-MDSCs) expressing CD11b^+^Ly6G^lo^Ly6C^hi^. As shown in **Figure 4G**, both PMN-MDSCs (CD11b^+^Ly6G^hi^Ly6C^lo^) and Mo-MDSCs (CD11b^+^Ly6G^lo^Ly6C^hi^) were present in the liver and spleen of mice treated with ConA+GSK872, and the majority of MDSCs were PMN-MDSCs.

### MDSCs mediate the hepatoprotective effect of GSK872

**Figure 5:**
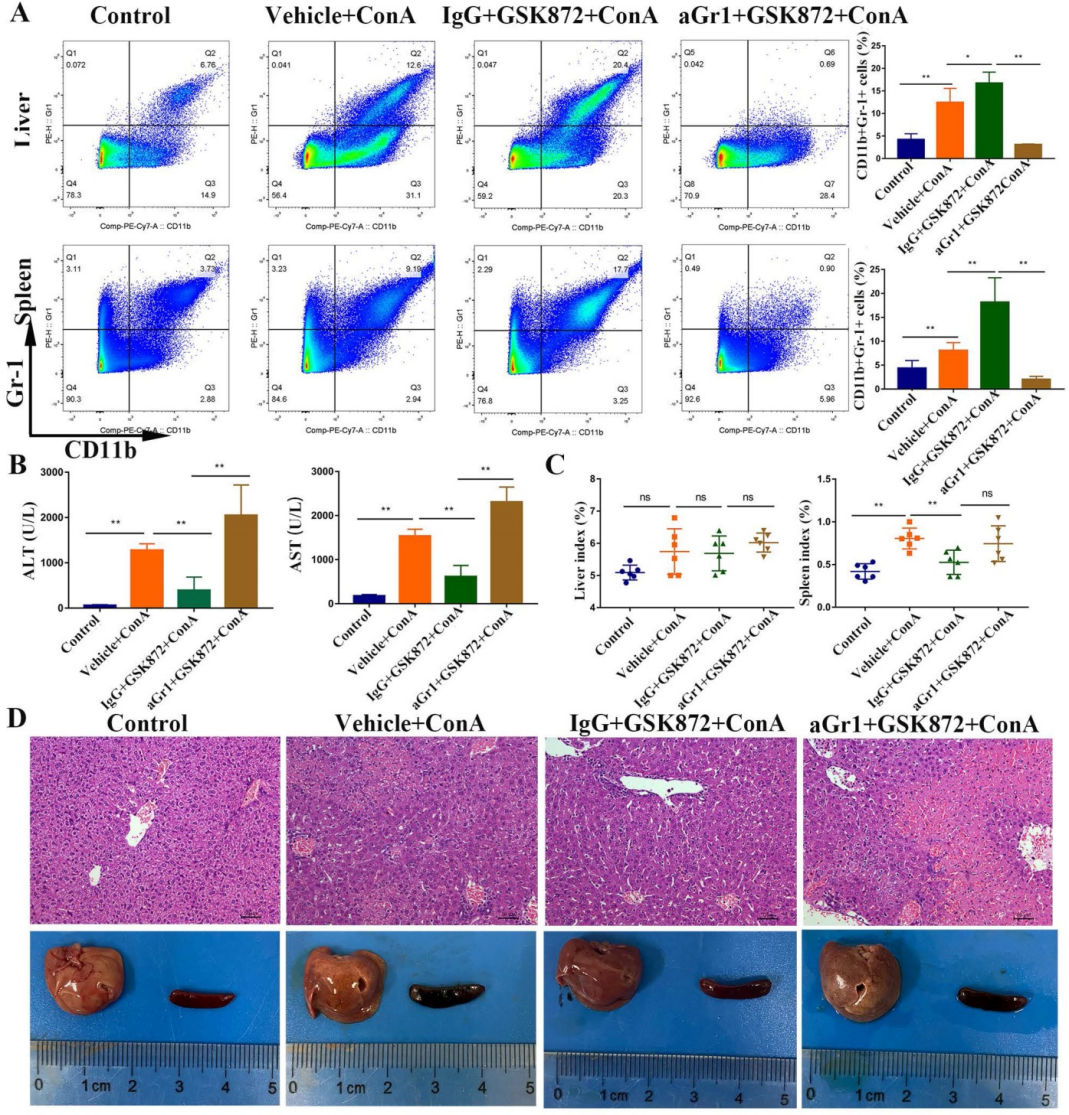
***In vivo* depletion of Gr-1 positive MDSCs abrogates GSK872-mediated hepatoprotection.** Mice were given anti-Gr-1 depleting antibody (250 µg) or control IgG (250 µg), treated with GSK872 or vehicle 36 h later, and followed by administration of ConA or PBS (n=6 per group). **(A)** Flow cytometry analysis of CD11b^+^Gr-1^+^ MDSCs in livers (up) and spleens (down). Representative scatter plots (left) are presented, and the histogram (right) represents the percentages of CD11b^+^Gr-1^+^ MDSCs.** (B)** Serum levels of ALT and AST were measured 12 h after ConA treatment. **(C)** Liver index (left) and spleen index (right) were determined 12 h after ConA treatment.** (D)** Representative photomicrographs (H&E staining; original magnification 200×; scale bars, 50 μm; up) and perusal images (down) of livers. All the values are shown as mean ± SD. ns., not significant; *P < 0.05, **P < 0.01.

Mice were given a depleting anti-Gr-1 antibody before GSK872 treatment to determine whether GSK872-mediated protection is mediated by MDSCs. The percentage of CD11b^+^Gr-1^+^ MDSCs increased after treatment with GSK872 and ConA, but the cells were virtually absent in hepatic mononuclear cells (HMNCs) and spleen cells isolated from mice pretreated with anti-Gr-1 and GSK872 and then injected with ConA **(Figure 5A)**. Furthermore, the percentage of both PMN-MDSCs and Mo-MDSCs (especially PMN-MDSCs) gated on CD45^+^CD11b^+^ MDSCs were significantly reduced in livers and spleens after anti-Gr-1 treatment (**Figure S2**), and the secondary antibody staining (antibody‐bound Gr-1^+^ MDSCs) was almost undetectable in livers and spleens (not shown). These results demonstrated the ability of anti-Gr-1 treatment to effectively deplete MDSCs in livers and spleens. Analysis of serum transaminases **(Figure 5B)**, spleen index **(Figure 5C)**, H&E staining of liver sections, and perusal images of livers and spleens **(Figure 5D)** showed that depletion of Gr-1^+^ MDSCs was accompanied by the lack of GSK872-mediated protective effect against ConA-induced immune hepatitis.

In order to confirm the hepatoprotective effect of MDSCs, we performed MDSCs-depletion experiments in ConA treated-mice (**Figure S3**). The results showed that both CD11b^+^Gr-1^+^ MDSCs and its subtypes (PMN-MDSCs and Mo-MDSCs) were markedly reduced in livers of aGr1+ConA group compared IgG+ConA group. In addition, analysis of serum ALT/AST and H&E staining of liver sections showed that depletion of Gr-1^+^ MDSCs was accompanied by more severe liver damage, confirming the effect of MDSCs on protection of ConA-induced injury.

### GSK872-induced anti-inflammatory effect requires the involvement of MDSCs

**Figure 6:**
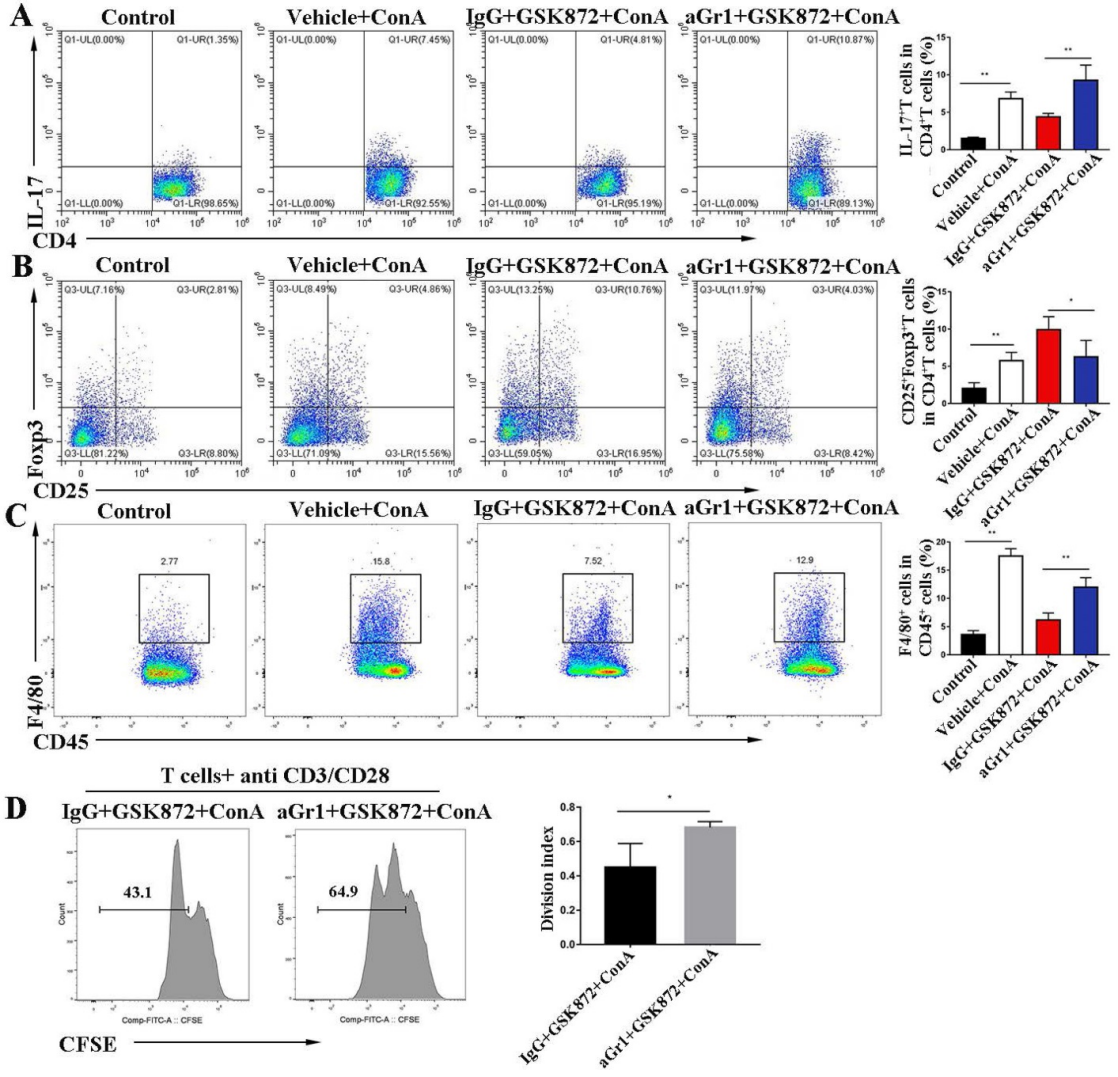
***In vivo* depletion of Gr-1 positive MDSCs attenuates GSK872-induced immunosuppression.** Flow cytometry analysis of CD4^+^IL-17^+^ cells **(A)** and CD4^+^CD25^+^Foxp3^+^ cells **(B)** in livers. Representative scatter plots (left) are presented, and the histogram (right) represents the percentages of CD4^+^IL-17^+^ cells and CD4^+^CD25^+^Foxp3^+^ cells in liver CD4^+^ T cells. **(C)** Flow cytometry analysis of CD45^+^F4/80^+^ macrophages in CD45^+^ HMNCs (left). The histogram (right) represents the statistical analysis of the percentages of CD45^+^F4/80^+^ macrophages in CD45^+^ HMNCs. **(D)** Splenic CD4^+^ T cells isolated from IgG+GSK872+ConA and aGr1+ GSK872+ConA treated-mice were cultured in the presence of anti-CD3/CD28 for 72 h. Flow cytometric analysis (left) and the statistical analysis (right) of the proliferation of CD4^+^ T cells. All the values are shown as mean ± SD. ns, not significant; *P < 0.05, **P < 0.01.

To investigate the mechanism by which Gr-1^+^ MDSC deletion abrogates the hepatoprotective effect mediated by GSK872, we examined the proinflammatory cells involved in IMH. Flow cytometry analysis showed that ConA-induced liver accumulation of CD4^+^IL-17^+^Th17 cells **(Figure 6A)** and CD45^+^F4/80^+^ macrophages **(Figure 6C)** was markedly reduced in mice pretreatment with GSK872. However, the depletion of Gr-1^+^ MDSCs dramatically reversed the GSK872-mediated low infiltration of CD4^+^IL-17^+^Th17 cells and CD45^+^F4/80^+^ macrophages and decreased the percentage of CD4^+^CD25^+^Foxp3^+^ Tregs **(Figure 6B)**. Furthermore, we isolated CD4^+^ T cells from the spleen of IgG+GSK872+ConA treated- or aGr1+GSK872+ConA treated-mice and analyzed their proliferation ability after stimulation with anti-CD3/CD28 for 3 days (**Figure 6D**). The results showed that anti-Gr-1 increased the proliferation ability of splenic CD4^+^ T cells compared to IgG control, indicating that MDSCs depletion promotes the activation of T cells. Thus, all these data indicate that MDSCs mediate GSK872-induced anti-inflammatory effect and immunosuppressive environment in livers of IMH mice.

### RIP3 blockade induces the production of chemokine (C-X-C motif) ligand 1 (CXCL1) in the liver

**Figure 7:**
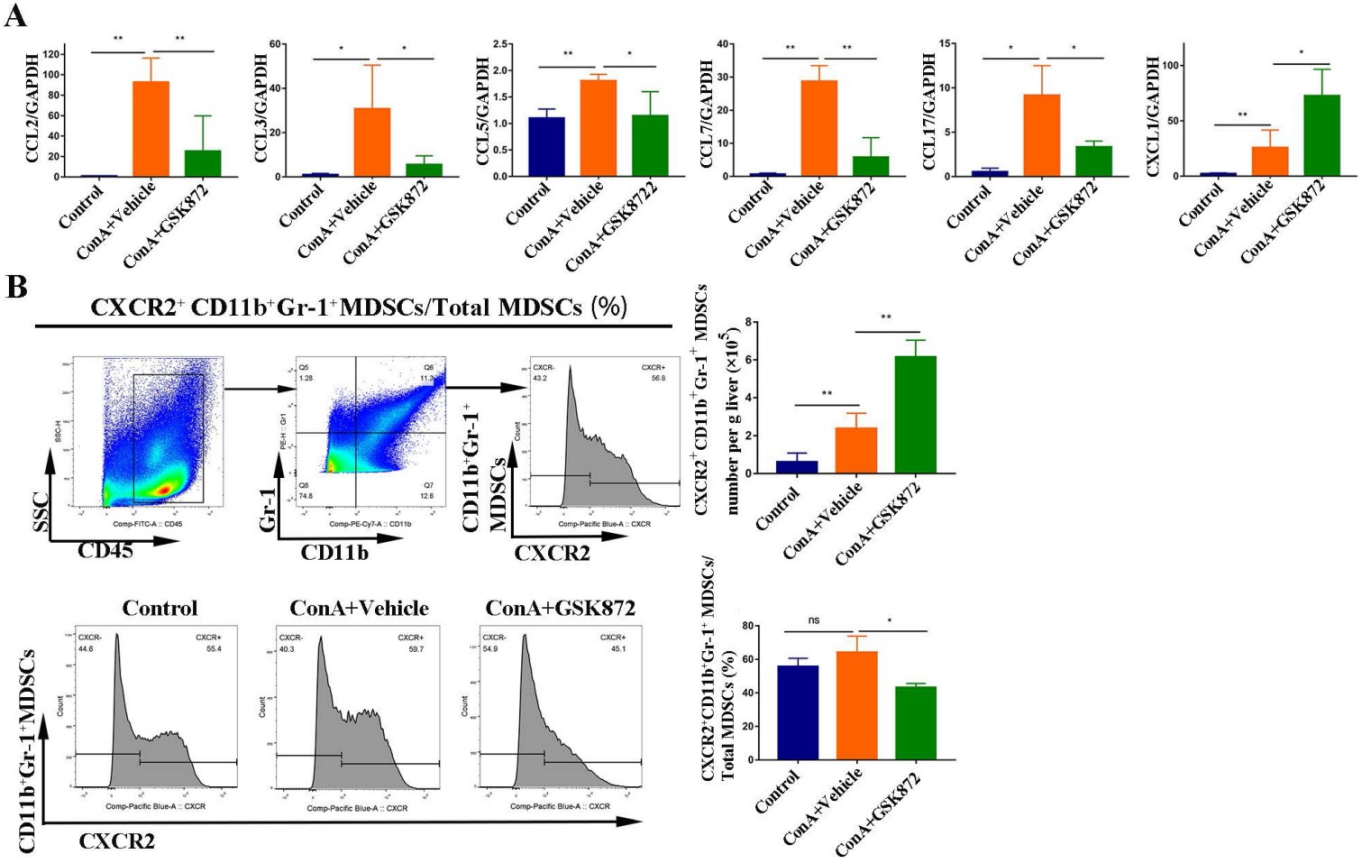
** GSK872 induces CXCL1 in the livers of ConA-treated mice.** Mice were given GSK872 or vehicle i.p. 1 h before treatment with ConA or PBS and sacrificed 12 h after ConA treatment. Livers were explanted and used for RNA and mononuclear cell extraction. **(A)** Gene expression levels of CCL2, CCL3, CCL5, CCL7, CCL17, and CXCL1 in liver tissues from mice treated with ConA and GSK872 were analyzed using RT-qPCR. Gene expression was normalized to GAPDH levels. **(B)** Flow cytometry analysis of the absolute cell number and percentage of CXCR2^+^CD11b^+^Gr^+^ MDSCs in livers. Gating strategies for CXCR2^+^CD11b^+^Gr-1^+^ MDSCs are shown (up). The histogram (right) represents the statistical analysis of the d absolute cell number of CXCR2^+^CD11b^+^Gr^+^ MDSCs in livers and the percentages of CXCR2^+^CD11b^+^Gr-1^+^ MDSCs in total liver CD11b^+^Gr-1^+^ MDSCs. Representative histograms in flow cytometry analysis (down) are presented. All the values are shown as mean ± SD. ns., not significant; *P < 0.05, **P < 0.01.

Chemotaxis and their cognate receptors play a crucial role in the migration of MDSCs into tissues 40; therefore, we identified the gene expression of several chemokines regulated by RIP3 inhibition **(Figure 7A)**. The expressions of C-C motif chemokine ligand (CCL)2, CCL3, CCL5, CCL7, and CCL17 all increased in the livers of mice treated with ConA but were markedly decreased by pretreatment with GSK872. By contrast, induction of IMH by ConA was accompanied by a significant upregulation of CXCL1 mRNA, and it was further increased by pretreatment with GSK872, consistent with the changes in the percentage of hepatic CD11b^+^Gr-1^+^ MDSCs.

To further delineate the possible crosstalk between CXCL1 and MDSCs, we next used flow cytometry to investigate the percentages of MDSCs expressing CXCR2, the cognate receptor for CXCL1. ConA significantly increased the absolute cell number of hepatic CD11b^+^Gr-1^+^ MDSCs expressing CXCR2 compared with the control mice. GSK872 further enhanced the absolute cell number of hepatic CD11b^+^Gr-1^+^ MDSCs expressing CXCR2 induced by ConA **(Figure 7B)**, consistent with the changes in the cell number of CD11b^+^Gr-1^+^ MDSCs in livers. Moreover, CD11b^+^Gr-1^+^ MDSCs isolated from HMNCs of the three groups all expressed a high level of CXCR2, indicating that CXCL1-CXCR2 axis contributes to MDSCs recruitment induced by RIP3 inhibition. Interestingly, although GSK872 further enhanced the cell number of hepatic CXCR2^+^CD11b^+^Gr-1^+^ MDSCs induced by ConA, the ratio of CXCR2^+^CD11b^+^Gr-1^+^ MDSCs to total CD11b^+^Gr-1^+^ MDSCs slightly reduced, suggesting that the CXCL1-CXCR2 axis is an important but not the only cause of the accumulation of liver MDSCs mediated by RIP3 inhibition.

## Discussion

The present study indicates that RIP3 blockade and subsequent MDSCs accumulation protected mice from ConA-induced immune hepatitis. RIP3 blockade reduced the production of proinflammatory cytokines and the infiltration of CD4^+^IL-17^+^ Th17 cells and CD45^+^F4/80^+^ macrophages through a MDSC-dependent mechanism, thus attenuating liver immune activity. Moreover, we showed that RIP3 inhibition may be a novel mechanism underlying the immunosuppressive effect of glucocorticoids on AIH. Therefore, RIP3 signaling can be a valuable therapeutic target in IMH.

RIP3, a member of the RIP kinase family, is a key inflammatory adapter and has critical roles in innate inflammation and immunoreaction through necroptosis and non-necroptosis function 41-44. The upregulation of RIP3 expression has been described in AIH and other immune disorders and supposed to contribute to the progression of the pathology 45, 46. Zhang et al. 31 showed that the accumulated macrophages express RIP3 in AIH liver tissues. And RIP3 deletion has been reported to protect against ConA-induced immune hepatitis 47. However, no investigation has been reported on the activation of RIP3 signaling and its role in liver inflammation during AIH development. In this study, we indicated that RIP3 and its effector molecule MLKL were activated in livers of AIH patients and ConA-induced immune hepatitis mice, and these expressions were localized in the majority of the macrophages (Kupffer cells). Blockading RIP3 signaling with GSK872, a specific RIP3 kinase inhibitor, prevented ConA-induced immune hepatitis and reduced the levels of transaminases and liver inflammation. Moreover, RIP3 signaling was demonstrated as a novel molecular mechanism of DEX treatment. Overall, these data indicate that targeting RIP3 signaling may be a valuable therapeutic strategy for the prevention of AIH.

Given that numerous immune cells (e.g., T cells, B cells, and macrophages) and cytokines are involved in the pathogenesis of AIH, we next explored the effect of RIP3 blockade on the production of proinflammatory cytokines and infiltration of immune cells in the livers of ConA-treated mice. Induction of immune hepatitis induced by ConA was followed by a significant upregulation of TNFα, IL-6, IL-1β, NLRP3, and IFNγ in liver tissues, whereas pretreatment of animals with GSK872 before administration of ConA caused a significant decrease in the expression of these proinflammatory cytokines. These cytokines are induced in AIH and contribute to the related detrimental immune reactions 27, 48. Moreover, we observed that RIP3 blockade modified the type of immune cells infiltrating the liver of IMH mice, decreasing the frequencies of CD4^+^IL-17^+^Th17 cells and CD45^+^F4/80^+^ macrophages but increasing those of CD4^+^CD25^+^Foxp3^+^ Tregs. The imbalance between Th17 and Tregs contributes to the induction and perpetuation of liver injury in AIH 12, 49. In addition, macrophages are present in dense portal cell infiltrates of AIH 50, whereas macrophage activation contributes to the inflammation and fibrosis in liver diseases 51, 52. These results show that RIP3 blockade attenuates liver immune activity response to ConA.

To dissect the mechanism of RIP3 blockade in regulating hepatic inflammatory reactions, we investigated the infiltration of MDSCs with significant immune inhibitory function in mice with IMH with or without GSK872 treatment. MDSCs have been implicated in the downregulation of immune responses through inhibiting the activation and proliferation of T cells, B cells, macrophages, and NK cells and reducing the production of proinflammatory factors 23, 53, 54. Diao et al. 27 and Liu et al. 54 demonstrated that the frequencies of MDSCs in mouse spleen and liver were increased at early stage of ConA treatment, MDSCs adoptive transfer protected mice from ConA-induced hepatitis, suggesting that the MDSCs might be involved in inhibiting early acute inflammation caused by ConA, increasing the frequency of MDSCs may be a novel strategy in suppressing liver inflammation. Consistent with these findings, we observed that MDSCs were expanded in the liver and spleen of ConA-induced mice, but reduced in the peripheral blood, implicating that the migration of periphery MDSCs to the liver and spleen may be involved in the initial resistance of mice against IMH. More importantly, we showed that RIP3 blockade can further promote the accumulation of MDSCs in liver, spleen, and periphery of mice with IMH. However, GSK872 by itself was not able to modify the percentage of MDSCs in liver and spleen of mice without hepatitis, it is not surprising. Because, it has been reported that inflammation is required for induction of MDSCs 55, 56. Although CD11b and Gr-1 are surface markers common to neutrophils and MDSCs, the immunosuppressive properties of MDSCs allow them to be distinguished from former 57. Hepatic MDSCs isolated from ConA+GSK872 and other group inhibited the proliferation of activated T cells *in vitro*, thus confirming their suppressive nature. Overall, these findings suggest that the immunosuppressive effect of RIP3 blockade may be associated with MDSCs expansion. To test the hypothesis, we evaluated the effect of MDSCs depletion on the effect of RIP3 blockade on the ConA-induced IMH. The depletion of MDSCs from mice abolished the GSK872-mediated protection against immune mediated liver damage and abrogated the GSK872-induced hepatic immune tolerance in IMH mice. Thus, RIP3 blockade protected mice from ConA-induced immune hepatitis through MDSC-mediated immune tolerance.

The underling mechanism by which GSK872 promotes accumulation of MDSCs in mice with IMH remains to be ascertained. Chemokines, including CCL2, CCL17, and CXCL1, play a crucial role in the recruitment of MDSCs into the tissues through their cognate receptors 37, 40. In this study, we showed that of all the chemokines evaluated, CXCL1 was one of the most abundantly expressed in ConA-treated mice and was further enhanced by RIP3 blockade, suggested that the expression of CXCL1 may be regulated by RIP3 kinase. The work of Li et al^58^ supports our assumption, they reported that RIP3 deficiency induced nuclear translocation of p-P65^Ser536^ and the binding of p-P65^Ser536^ to the CXCL1 promoter increased the transcription of CXCL1. By contrast, the expressions of CCL2, CCL3, CCL5, CCL7, and CCL17 increased in the livers of mice treated with ConA but were downregulated by GSK872 treatment. Consistently, CD11b^+^Gr1^+^ MDSCs isolated from livers of mice with or without ConA treatment expressed high levels of CXCR2, the CXCL1 cognate receptor. Although GSK872 further enhanced the cell number of hepatic CXCR2^+^CD11b^+^Gr-1^+^ MDSCs induced by ConA, the ratio of CXCR2^+^CD11b^+^Gr-1^+^ MDSCs to total CD11b^+^Gr-1^+^ MDSCs slightly reduced, suggesting that the CXCL1-CXCR2 axis is an important but not the only cause of the accumulation of liver MDSCs mediated by RIP3 inhibition. Further studies are required to determine whether RIP3 inhibition converts tissue resident cells into MDSCs.

In conclusion, our study demonstrated for the first time that MDSCs accumulation derived by RIP3 blockade can effectively protect mice from ConA-induced IMH through downregulating hepatic inflammatory responses, raising the possibility that RIP3 blockade can be a promising new therapeutic approach for treating AIH.

## Supplementary Material

Supplementary figures.

## Figures and Tables

**Figure 8:**
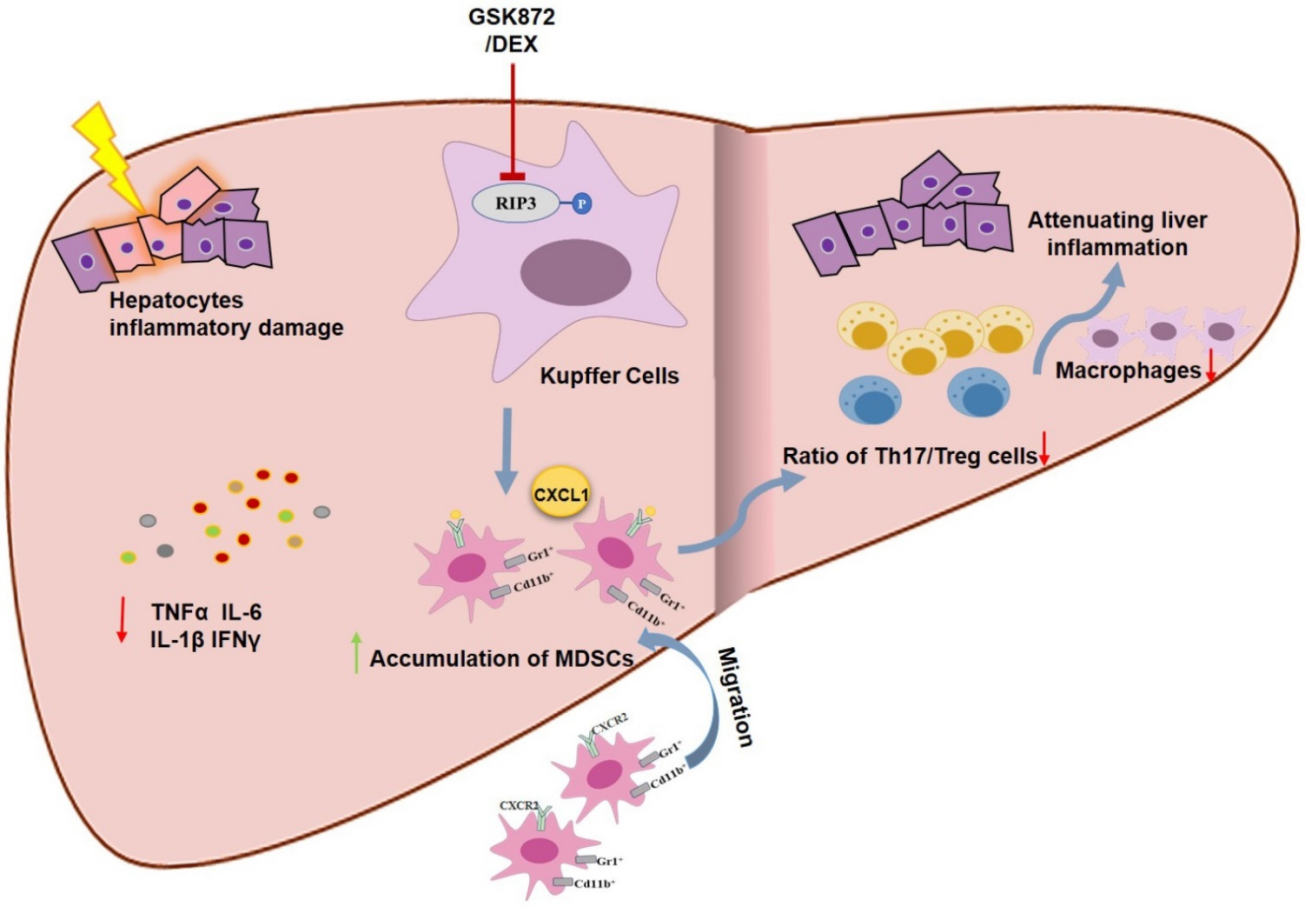
Schematic representation of RIP3 blockade in preventing immune-mediated hepatitis. RIP3 kinase inhibition promotes the hepatic accumulation of MDSCs by CXCL1-CXCR2 axis and other unknown factors. Accumulated MDSCs mediate the hepatoprotective effect of GSK872 by reducing the frequencies of Th17 cells and macrophages but increasing those of Treg cells.

**Table 1:** The primer sequences used in RT-qPCR

Gene	Forward Sequence	Reverse Sequence
RIP3	GAAGACACGGCACTCCTTGGTA	CTTGAGGCAGTAGTTCTTGGTGG
MLKL	CTGAGGGAACTGCTGGATAGAG	CGAGGAAACTGGAGCTGCTGAT
TNFα	GAAGTTCCCAAATGGCCTCC	GTGAGGGTCTGGGCCATAGA
IL-6	ACAACCACGGCCTTCCCTACTT	CACGATTTCCCAGAGAACATGTG
IL-1β	CTTTGAAGTTGACGGACCC	TGAGTGATACTGCCTGCCTG
NLRP3	TCACAACTCGCCCAAGGAGGAA	AAGAGACCACGGCAGAAGCTAG
IFNγ	CGGCACAGTCATTGAAAGCCTA	GTTGCTGATGGCCTGATTGTC
iNOS	TCTAGTGAAGCAAAGCCCAAC	TGGCCTTGTGGTGAAGAGTG
CCL2	AGCTGTAGTTTTTGTCACCAAGC	GTGCTGAAGACCTTAGGGCA
CCL3	CCCAGCCAGGTGTCATTTTC	GTGGCTACTTGGCAGCAAAC
CCL5	TGCTGCTTTGCCTACCTCTC	TCCTTCGAGTGACAAACACGA
CCL7	CGCTGCTTTCAGCATCCAAG	CTTGAAGATAACAGCTTCCCAGG
CCL17	CAATGTAGGCCGAGAGTGCTG	GCATCCCTGGAACACTCCACTG
CXCL1	CTGCACCCAAACCGAAGTC	AGCTTCAGGGTCAAGGCAAG
GAPDH	GGCAAATTCAACGGCACAGT	CGCTCCTGGAAGATGGTGAT

## Abbreviations

AIH: autoimmune hepatitis
RIP3: receptor interacting protein kinase 3
ConA: concanavalin A
MDSCs: myeloid-derived suppressor cells
Tregs: T regulatory cells
NK: natural killer
IMH: immune-mediated hepatitis
TNF: tumor necrosis factor
MLKL: mixed lineage kinase domain like protein
IL: interleukin
NLRP3: NLR family pyrin domain containing 3
IFN: interferon
iNOS: inducible nitric oxide synthase
CCL: C-C motif chemokine ligand
i.v.: intravenously
i.p.: intraperitoneally
DEX: dexamethasone
PBS: phosphate-buffered saline
ALT: alanine aminotransferase
AST: aspartate aminotransferase
HMNCs: hepatic mononuclear cells
CXCL1: chemokine (C-X-C motif) ligand 1
CXCR2: chemokine (C-X-C motif) receptor 2
FITC: fluorescein isothiocyanate
SD: standard deviation
