# Supplementary material for: RIP3 blockade prevents immune-mediated hepatitis through a myeloid-derived suppressor cell dependent mechanism
Source: Int J Biol Sci. 2022 Jan 1;18(1):199–213. doi: 10.7150/ijbs.65402 (PMC8692153; doi:10.7150/ijbs.65402)
Supplement: Supplementary file 1 — Supplementary figures. [file ijbsv18p0199s1.pdf]

Supplementary Material

Supplementary FIGURE LEGENDS

Figure S1 | GSK843 induces the accumulation of $CD11b^+Gr\text{-}1^+$ cells in the liver and spleen of ConA-treated mice. Mice were pre-treated i.p. with GSK843 (0.5mg/kg; MCE, USA) or vehicle 1 hour before ConA (15 mg/kg) administration; mice were sacrificed 12 hours later after ConA injection and livers and spleen were collected. Representative dot-plots (left) and the histograms (right) show the percentages of $CD11b^+Gr\text{-}1^+$ cells in HMNCs and spleen cells of mice treated with GSK843+ConA or ConA alone. All the values are shown as mean ± SD. ns., not significant; *P < 0.05, **P < 0.01.

Figure S2 | The effect of B6-8C5 antibody on $Gr\text{-}1^+$ MDSCs depletion. Hepatic mononuclear cells (HMNCs) and spleen cells from IgG+GSK872+ConA treated- and aGr1+GSK872+ConA treated-mice were assayed by FACS. (A) Percentages of $CD11b^+Ly6G^{hi}Ly6C^{lo}$ cells (PMN-MDSCs) and $CD11b^+Ly6G^{lo}Ly6C^{hi}$ (Mo-MDSCs) cells in $CD45^+CD11b^+$ cells were analyzed by flow cytometry. The percentages (up) and absolute cell numbers (down) of PMN-MDSCs and Mo-MDSCs in HMNCs (B) and spleen cells (C). All the values are shown as mean ± SD. ns., not significant; *$P < 0.05$, **$P < 0.01$.

Figure S3 | In vivo depletion of Gr1-positive MDSCs aggravates ConA induced liver injury. Mice were given anti-Gr1 depleting antibody (250μg/ mice) or control IgG (250μg) 36 hours before ConA(15mg/kg) treatment, mice were sacrificed 12 hours after ConA-treatment and blood and liver samples were collected. (A) Percentages of $CD11b^+Gr\text{-}1^+$MDSCs and its subtypes (PMN-MDSCs and Mo-MDSCs) cells in livers were analyzed by flow cytometry. (B)The Serum levels of ALT/AST in two groups. (C) Representative photomicrographs (H&E staining; original magnification 200×; scale bars, 50 um) of livers in two groups. All the values are shown as mean ± SD. ns., not significant; *$P < 0.05$, **$P < 0.01$.

Figure S1

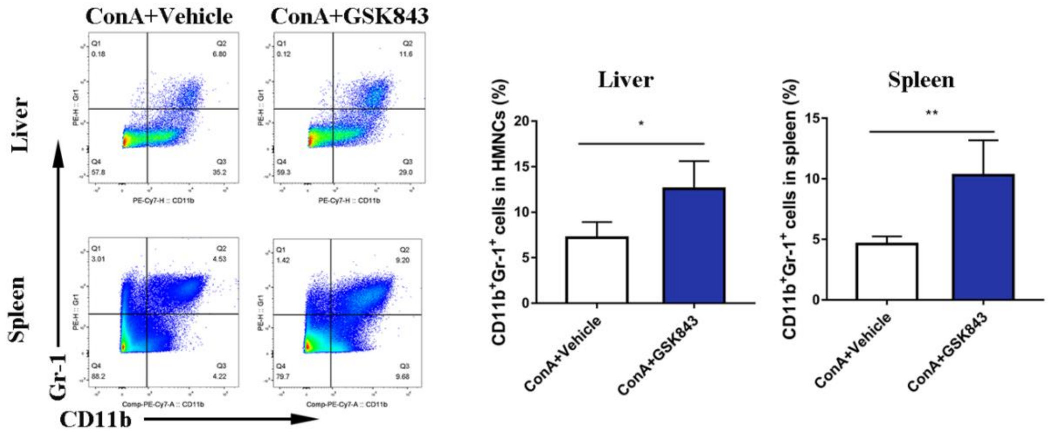
ConA+Vehicle
ConA+GSK843
Liver
Spleen
Gr-1
CD11b
Liver
Spleen
CD11b+Gr-1+ cells in HMNCs (%)
CD11b+Gr-1+ cells in spleen (%)
ConA+Vehicle
ConA+GSK843
*
**

Figure S2

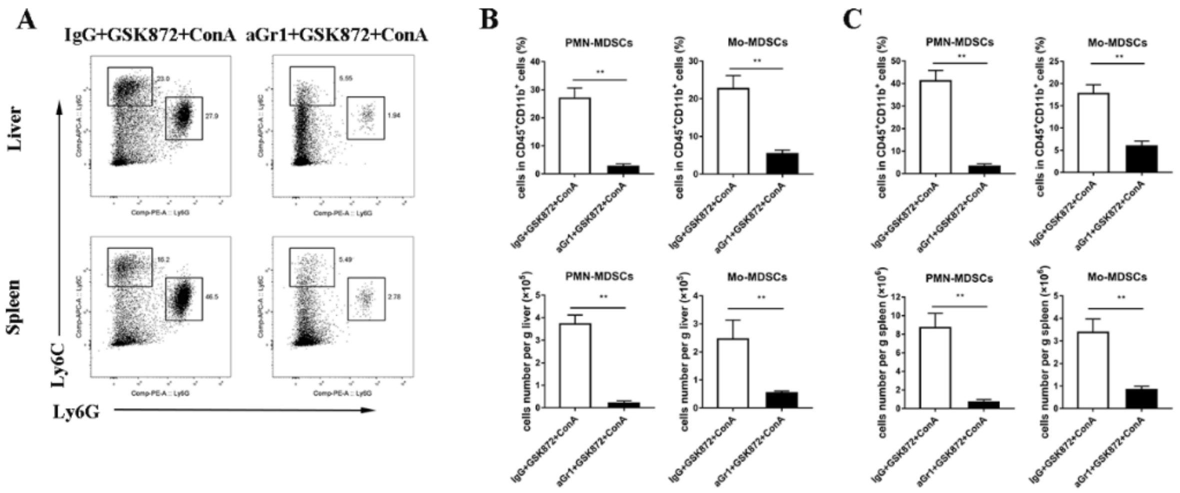
A
IgG+GSK872+ConA
aGr1+GSK872+ConA
Liver
Spleen
Ly6C
Ly6G
B
C
PMN-MDSCs
Mo-MDSCs
cells in CD45+CD11b+ cells (%)
IgG+GSK872+ConA
aGr1+GSK872+ConA
**

Figure S3

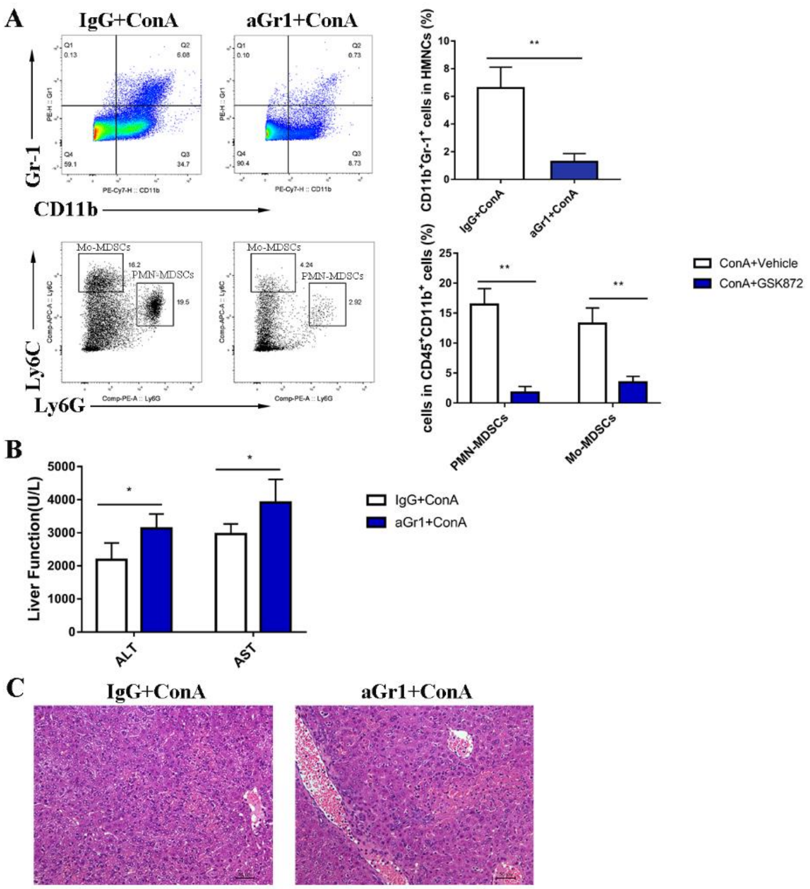
A
IgG+ConA
aGr1+ConA
Gr-1
CD11b
Ly6C
Ly6G
Mo-MDSCs
PMN-MDSCs
CD11b+Gr-1+ cells in HMNCs (%)
IgG+ConA
aGr1+ConA
cells in CD45+CD11b+ cells (%)
PMN-MDSCs
Mo-MDSCs
ConA+Vehicle
ConA+GSK872
B
Liver Function(U/L)
ALT
AST
IgG+ConA
aGr1+ConA
C
IgG+ConA
aGr1+ConA
